# Genomic Size Is Critical to Guarantee the Genomic Stability of Non-Replicative HSV1 Vectors

**DOI:** 10.3390/ijms26104941

**Published:** 2025-05-21

**Authors:** Justine Basset, Alexandra Seraffin, Julien Ratelade, Yohann Dickx, Tomasz Benedyk, Grzegorz Sarek, Teddy Jégu, Alberto L. Epstein

**Affiliations:** EG427, 29 Rue du Faubourg Saint Jacques, 75014 Paris, France

**Keywords:** non-replicative HSV-1 vectors, genomic stability, genome size, stuffer DNA

## Abstract

Non-replicative herpes simplex virus type 1 (nrHSV-1) vectors are promising delivery vehicles for gene therapy due to their large DNA payload capacity and ability to infect a broad range of cell types. However, the genomic deletions made to generate such nrHSV-1 vectors can result in undersized genomes that trigger genomic instability—including rearrangements and size extensions—compromising their therapeutic potential. This study investigates the stabilization of undersized nrHSV-1 vectors through the insertion of stuffer DNA segments. We assess genomic stability, productivity, toxicity, and transgene expression in vitro and in vivo. Our findings demonstrate that nrHSV-1 can accommodate variations in genome size up to 5–6% and highlight the importance of maintaining a genome size close to that of the wild-type HSV-1 for enhanced genomic stability and sustained transgene expression without adverse effects. This strategy offers a promising approach for optimizing nrHSV-1 vectors for clinical applications.

## 1. Introduction

Many icosahedral viruses exhibit a strong preference for precise genome sizes. Altering this preferred size disrupts genome stability and can negatively impact adenovirus vector productivity [1]. The mechanisms by which nucleic acid content contributes to viral genome stability likely vary depending on the virus and the nature of its genetic material (e.g., dsDNA, ssDNA, or RNA). Consistency across manufacturing lots is crucial, particularly in the late stage of development where genome size variability can compromise virion stability and vector quality, leading to batch-to-batch inconsistencies. In viral gene therapy, such variations can affect both vector stability and therapeutic efficacy, underscoring the need for stringent quality control and process optimization to ensure the reliable production of high-quality vectors.

Previous studies on adeno-associated virus (AAV) vectors have shown that genomes reduced to less than 80% of their wild-type (WT) length often undergo genomic rearrangements to restore a size closer to the WT genome [2]. While genome stability can usually be maintained at around 90% of the WT length of the AAV, optimal vector stability and high yield during manufacturing are best achieved when the genome size is maintained near 100% of the WT length of the adeno-based vector [3,4]. However such thresholds seem to be virus-dependent.

In AAV vectors, the genome size significantly impacts the packaging efficacy due to the physical and biological constraints of the viral capsid [2,5]. Each viral vector has a specific minimum and maximum genome size necessary for proper capsid assembly. Overloading the capsid with genetic material can lead to instability, incorrect assembly, or degradation of the vector. Incomplete or fragmented genomes may be packaged, reducing the efficiency of gene delivery. Conversely, if the genome is too small, the capsid may form improperly or become structurally unstable, resulting in defective viral particles. Additionally, too small a genome may cause the virus to package unwanted host, plasmid DNA, or a partially duplicated viral genome, leading to variability in gene transfer [6,7].

Viral packaging mechanisms often depend on recognizing specific sequences or structural signals in the genome. Deleting large portions of the viral genome can disrupt the spacing or positioning of these signals, reducing the likelihood of proper encapsidation and lowering production yields due to inefficient packaging [8]. Furthermore, an inadequate genome size can lead to a higher proportion of empty capsids [5], which are non-functional and more likely to be cleared by the host immune system [9].

Over the past 30 years, significant progress has been made in the development of gene delivery vectors based on the herpes simplex virus 1 (HSV-1), with HSV-1-based vectors emerging as promising tools for gene therapy in the treatment of various diseases [10,11,12,13,14,15,16]. HSV-1 is an enveloped virus containing a 152 kbp linear double-stranded DNA genome arranged in long and short unique segments (UL and US), flanked by inverted repeat sequences (Terminal Repeat Long TRL/Internal Repeat Long IRL and Internal Repeat Short IRS/Terminal Repeat Short TRS). The HSV-1 genome is encapsulated in an icosahedral capsid and encodes more than 80 genes, which can be classified as essential or non-essential based on their requirement for viral replication and production in vitro [17]. Non-replicative HSV-1 vectors (nrHSV-1) are created by deleting essential viral genes, rendering them incapable of producing infectious progeny while still retaining the ability to deliver therapeutic transgenes to target cells. These vectors can accommodate large genetic payloads, making them suitable for the delivery of complex gene-editing tools, large cDNA sequences, full genomic locus, or multiple therapeutic genes. Moreover, HSV-1 vectors can persist episomally in the nucleus of non-dividing cells, which reduces the risk of insertional mutagenesis compared to integrating viral vectors [18,19].

To develop a safe, non-toxic, and nrHSV-1-based DNA delivery platform for genetic medicine, large portions of the viral genome must be deleted. However, these deletions often result in a vector genome size below 90% of the wild-type genome size [1,20,21,22]. The impact of such large deletions on the nrHSV-1 vector genome’s stability is not well understood. The recent advent in long-read DNA sequencing technology allows us to investigate for the first time, to our knowledge, the relationship between nrHSV-1 vector genome size and its genomic stability. We analyzed viral vectors with varying genome sizes resulting from deletions of essential and non-essential viral genes. To maintain the vector genome size within the optimal limits for efficient DNA packaging—ideally close to 100% of the WT genome length—these deletions were compensated by inserting “stuffer” sequences composed of non-coding HSV-1 DNA with a GC content similar to that of the WT virus. Our findings provide critical insights into how genome size affects the stability of nrHSV-1 vectors, with important ramifications for enhancing vector design in gene therapy applications.

## 2. Results

### 2.1. Genomic Stability of Non-Replicative HSV-1 Vector Depends on the Net Genome Size

**Figure 1:**
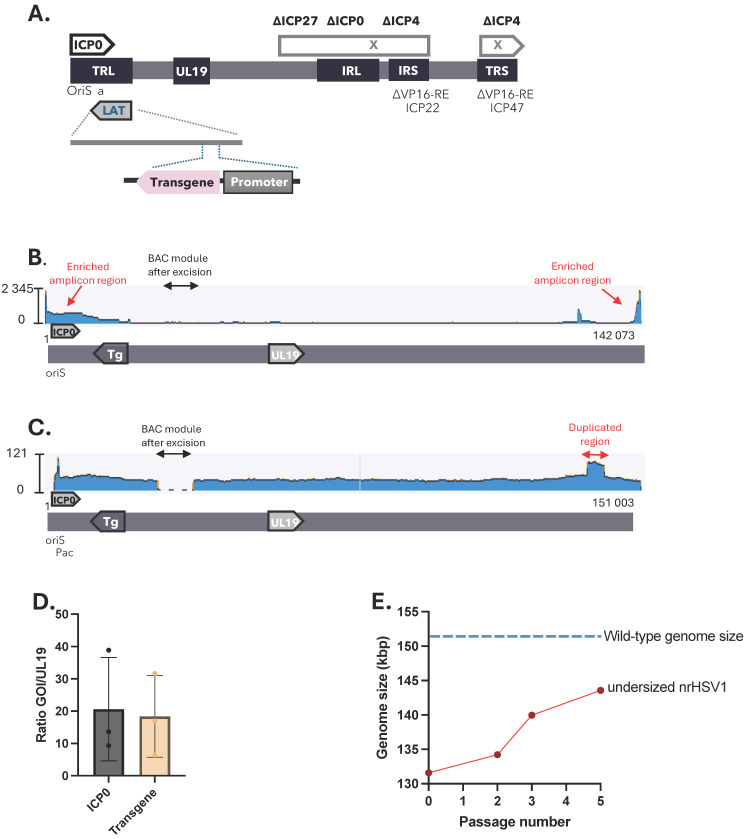
Undersized nrHSV-1 genomes undergo genomic rearrangements and size extension overtime. (**A**) Schematic representation of nrHSV1 vector genome. The vector is deleted for the joint region, spanning from ICP27 IE gene to the second copy of ICP4 IE gene (Δ; gray boxes). Additionally, the VP16-responsive elements (VP16-RE) located in both ICP22 and ICP47 IE promoter have been deleted (ΔVP16-RE). The vector contains an expression cassette for reporter transgene in the LAT region. (**B**,**C**) Representative Oxford Nanopore Technology long-read sequencing of nrHSV1 vector genomic DNA extracted from purified vector stocks. The coverage is represented as a blue line with read depth (measured in the number of reads per base) plotted along the y-axis. Localisation of relevant genomic elements are shown (origin of replication (OriS), packaging signal (pac), ICP0, transgene (Tg), UL19 gene). Recombination by products amplicon populations (**B**) and genome DNA region duplication (**C**) are indicated with red arrows. (**D**) Relative viral gene copy in nrHSV1 genome. nrHSV1 vector was amplified in Vero 7b cells at low MOI. Viral genomes were extracted from purified vector stocks and relative ICP0 gene or transgene copy numbers were determined by qPCR and normalized to the UL19 gene. GOI means gene of interest. (**E**) Undersized nrHSV1 vector genome size increases over passages. Serial passages of undersized nrHSV1 in Vero 7b cells at low MOI were performed. The genomic size (kbp) of purified nrHSV1 stocks was evaluated by ONT sequencing.

Large portions of the HSV-1 genome had to be deleted to construct a reliable, non-toxic, and non-replicative HSV-1 (nrHSV-1) delivery vehicle for genetic medicine. To that end, the joint region spanning from the ICP27 to ICP4 IE genes—including, among others, one copy of ICP0, as well as the second copy of the ICP4 gene—were deleted. In addition, both promoters of ICP22 and ICP47 IE genes were deleted for VP16-responsive elements to minimize viral gene expression and potential toxicity risk. The vector contains one expression cassette, inserted in the LAT region, for the expression of a reporter transgene under a non-viral promoter (Figure 1A).

While the deletion of sequences within the nrHSV-1 genome provides space for payload insertion, extensive deletions result in the viral vector genome size decreasing by up to 15% (minimal genome size of 131,553 bp). To investigate whether nrHSV-1 vector genome stability depends on net genome size, we conducted sequencing experiments on nrHSV-1 vectors that differed in genome size. Oxford Nanopore Technology (ONT) long-read sequencing of an nrHSV1 vector with an original genomic size of 131,553 bp revealed that the resulting vector underwent rapid rearrangements during the serial passages in cell culture. This manifested as genomic instability of the virus population, giving rise to a high degree of genomic variability such as the spontaneous generation of amplicons (Figure 1B, Supplementary Figure S1), nucleotide substitution, and/or duplicated genome regions (Figure 1C). Amplicons, large concatemeric genomes with repetitive units of limited complexity (see [23] and Supplementary Figure S1), were found in viral stocks with unit sizes ranging from ~1.5 kb to ~15 kb. These amplicons always contained one origin of DNA replication (oriS) and the packaging signal (pac). Amplicons might also include the ICP0 IE gene, which can induce cytotoxic effects when overexpressed [24], and the transgene resulting in vector batch variability (Figure 1B,C, Supplementary Figure S2).

To confirm the sequencing data, the gene copy number was evaluated by qPCR by targeting three independent genes. The major capsid gene UL19, known for its high stability and located far away from both oriS regions, was used as a reference gene, while the ICP0 gene (close gene to oriS) and transgene (inserted within the LAT region) copy number should reflect the presence of amplicons or duplication within this region. We observed high variability in terms of the ICP0 gene and transgene copy number ranging from 5 to 38 when compared to UL19, thus confirming the presence of genomic rearrangements that contribute to the variability in genome size (Figure 1D). Similarly to duplications, the presence of amplicons (which, due to their concatemeric nature, are vastly overrepresented in the sequencing data: >90% of aligned reads), most likely represents a form of genomic instability due to a suboptimal (small) vector size. Interestingly, the undersized nrHSV-1 viral genome showed progressive enlargement, evidenced by increasing net genome size and the frequency of rearrangements over successive passages (Figure 1E). Therefore, these undesired recombination events prevented the generation of homogeneous vector stocks. These findings suggest that there is a relatively tight constraint on the size of DNA that can be packaged into virions, and that decreasing the genome size of the nrHSV-1 vector results in a sharp decrease in the viral genome’s stability.

### 2.2. Stuffed nrHSV-1 Vectors Have Higher Productivity

**Figure 2:**
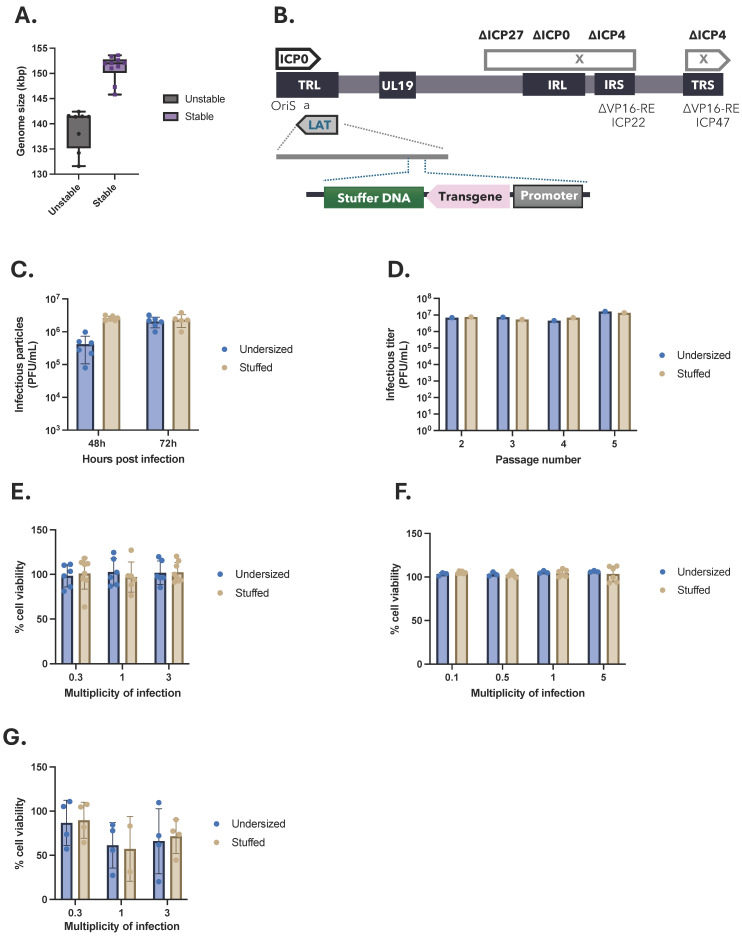
Stuffed nrHSV-1 vectors have higher productivity. (**A**) Genome size distribution among stable and unstable nrHSV-1 vectors. Vectors with varying genome lengths were amplified in Vero 7b cells and viral genomes were screened for genomic rearrangements using long read ONT-sequencing. Vectors presenting rearrangements were classified as unstable while vectors without any rearrangement were identified as stable. (**B**) Schematic representation of nrHSV1 vector genome containing the stuffer DNA sequence inserted in the LAT region downstream the transgene polyadenylation sequence. (**C**) Viral vector growth curve. nrHSV1 vectors containing or not the stuffer sequence were propagated in complementing Vero 7b cells at low MOI. Levels of infectious particles produced at 48 h or 72 h pi were measured by plaque forming unit assay. (**D**) Viral vector productivity over passages. Undersized or stuffed nrHSV1 were propagated in complementing Vero 7b cells for up to 5 successive passages at MOI 0.03. Samples were collected at 72 h pi and infectious particles were quantified by plaque assay. (**E**–**G**) Toxicity induced by nrHSV-1 infection. Dividing Vero cells (**E**) and Neuro 2a cells (**F**) were infected with increasing MOI of nrHSV1 vectors ± stuffer sequence. Cell viability was assessed 24 h pi by luminescent-based assay. Non dividing cells iPSC-derived glutamatergic neurons were infected with increasing MOI of nrHSV1 vectors ± stuffer sequence (**G**) Cell viability was assessed 48 h pi by luminescent-based assay.

An unbiased comparison of genome size distribution among nrHSV-1 vectors with varying genomic lengths revealed that nrHSV-1 vectors with a genome size smaller than 143 kbp (94% of WT genome) exhibited genomic rearrangements after only two to four passages, indicating poor genomic stability (Figure 2A). In contrast, nrHSV-1 vectors with a genome size of 95% of the wild-type genome (larger than 145 kbp) showed no genomic rearrangements up to 10 passages, suggesting these vectors remained stable over time (Figure 2A). These findings suggest that developing an nrHSV-1 vector with a genome size varying less than 5% from the wild-type is critical to maintaining genomic stability during serial passages.

To maintain the genome homogeneity of nrHSV-1 vectors, we have filled in the size gap between the genomic deletions and the insertion of the desired transgene with a “stuffer DNA” to reach at least 97% of the wild-type genome size (147.5 kbp) to avoid genomic instability. To this end, we have designed a stuffer DNA fragment based on short non-coding sequences from the HSV-1 genome, which were randomly assembled into a 20 kbp sequence with similar GC content as the rest of the viral genome. In addition, the stuffer DNA sequence upon design was screened to not contain any open reading frames (ORFs) or promoter regions using Geneious Prime software. This stuffer DNA was then introduced into the genome structure of the nrHSV-1 vectors, downstream from the transgene polyadenylation sequence in the LAT region (Figure 2B). Long-read ONT sequencing confirmed the successful incorporation of the stuffer DNA (Supplementary Figure S3). The resulting vector, called the stuffed vector, reaches a net genome size closer to that of the wild-type virus (153,587 bp). Because the insertion of this sequence is located close to the region containing the oriS and packaging sequences, we ensured that the introduction of such a large non-coding DNA sequence did not impair the viral replication or packaging efficiency. Thus, we assessed whether the stuffed nrHSV-1 vector could generate stable high-titer stocks over multiple passages. To that end, Vero 7b cells were infected with an undersized vector (138,288 bp) or a stuffed vector (153,213 bp) at a low multiplicity of infection (MOI 0.03). Samples were collected at 48 h and 72 h post-infection and infectious viral titers were measured using a plaque-forming-unit assay. While the nrHSV-1 vectors with an undersized genome display a peak of productivity at 72 h post-infection, the stuffed vector reaches an equivalent productivity level earlier at 48 h post-infection (Figure 2C). These observations suggest that the nrHSV-1 vector bearing a stuffer DNA is more efficiently packaged and, therefore, more infectious compared to the undersized vector. In addition, similar infectious titers were obtained for both vectors over successive passages (up to five passages) in complemented Vero 7b cells infected at a low multiplicity of infection (Figure 2D). These observations mean that the introduction of a stuffer DNA in the genome of nrHSV-1 did not alter its replication and production yields in complementing Vero 7b cells. Better yet, restoring the genomic size close to the wild type improves viral productivity by reducing the time taken to reach the productivity peak.

To ensure the stuffer region does not induce cytotoxicity of the nrHSV1 vector upon transduction of non-complementing cells, we measured the cell viability in vitro. Dividing Vero or Neuro2a cells—known to be sensitive to HSV-1 vector toxicity [20]—were infected with both types of nrHSV-1 vectors at increasing MOI values for 24 h. Cellular ATP levels, which directly correlate with the number of metabolically viable cells, were determined using a luminescent-based assay. No significant change in luminescence was observed between the two vectors at all tested MOI values (Figure 2E,F), suggesting that the stuffer DNA insertion did not induce cytotoxicity compared to the vectors with an undersized genome. Interestingly, similar results were obtained in non-dividing cells. Indeed, iPS cell-derived cortical glutamatergic neurons infected with increasing MOI values of the stuffer vector show stable cellular ATP-level at 2 days post-infection (Figure 2G). These data indicate that the transduction of dividing or non-dividing cells with an nrHSV-1 vector bearing a stuffer DNA in its genome did not impair cellular viability.

### 2.3. Stuffed nrHSV-1 Vectors Display Genomic Stability over Time

**Figure 3:**
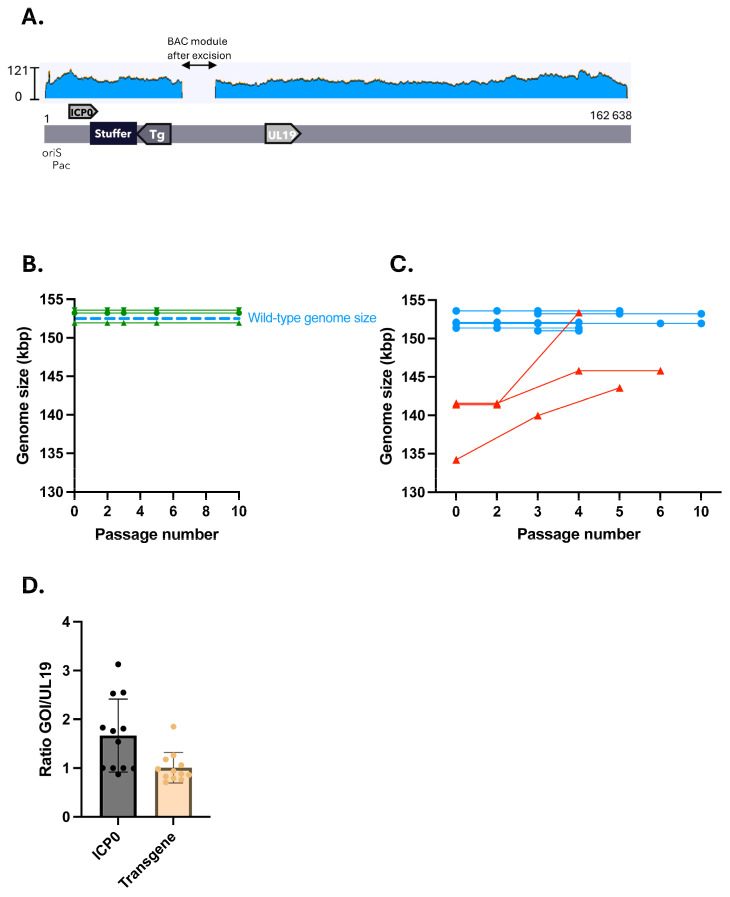
Stuffed nrHSV-1 vectors display genomic stability over time. (**A**) Representative Oxford Nanopore Technology sequencing of the stuffed nrHSV1 vector. Genomic DNA were extracted from purified vector stocks. The coverage is represented as a blue line with read depth (measured in the number of reads per base) plotted along the y-axis. Localisation of relevant genomic elements are shown (origin of replication (OriS), packaging signal (pac), ICP0, stuffer DNA, transgene (Tg), UL19 gene) (**B**) Stuffed nrHSV1 vector genome size are stable over passages. Serial passages of stuffed nrHSV1 vectors in complementing Vero 7b cells at low MOI were performed. The genomic size (kbp) of purified nrHSV1 stocks were evaluated by ONT sequencing. (**C**) nrHSV1 vectors genome size over passages. Serial passages of undersized or stuffed nrHSV1 vectors in Vero 7b cells at low MOI were performed. The genomic size (kbp) of purified nrHSV1 stocks were evaluated by ONT sequencing. Stuffed vectors are presented in blue lines. Undersized vectors are shown in red lines. (**D**) Relative viral gene copy in the genome of a stuffed nrHSV1 vector. nrHSV1 vectors containing the stuffer sequence DNA were amplified in Vero 7b cells at low MOI. Viral genomes were extracted from purified vector stocks and relative ICP0 gene or transgene copy numbers were determined by qPCR and normalized to the UL19 gene.

To assess whether the insertion of the stuffer DNA sequence into the viral vector genomes and restoration of the optimal genome size allowed for more genomic stability, we carried out vector production through several passages in complementing Vero 7b cells. Viral vector genomes were then analyzed by ONT long-read sequencing. We did not observe the formation of complex genomic rearrangements—such as amplicons or duplicated regions of the viral genome—in vectors with stuffer DNA (Figure 3A and Supplementary Figure S3). Analysis of the stuffed vectors’ net genome size showed no variation in length over up to 10 passages in the Vero 7b cells, demonstrating the maintenance of genomic stability in contrast to the undersized vectors (Figure 3B,C). In addition, consistent viral gene copy numbers among stuffed nrHSV-1 vectors compared to their undersized counterparts were also found by qPCR analysis (Figure 1D and Figure 3D). Taken together, these data indicate that the stuffer DNA reduced the occurrence of genomic rearrangements and duplications, thereby stabilizing the vectors.

### 2.4. Insertion of the Stuffer DNA in nrHSV1 Vector Genome Does Not Impact Transgene Expression

**Figure 4:**
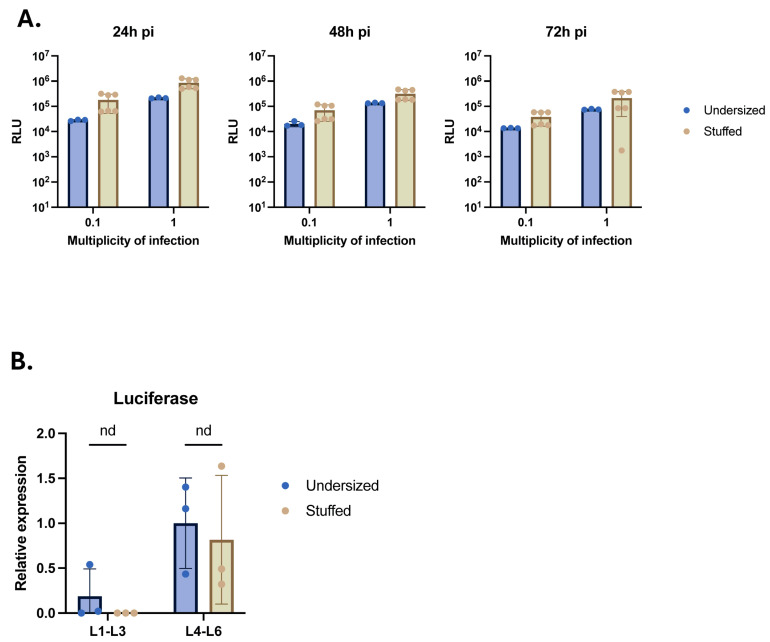
Reporter transgene expression is not impact by the DNA stuffer sequence. (**A**) Luciferase activity (RLU) in infected non-complementing Vero cells. Cells were infected with nrHSV1 ± stuffer DNA sequence at the indicated MOI and luciferase activity was measured at 24 h, 48 h and 72 h pi. (**B**) Luciferase transgene expression. mRNAs were extracted from pooled L1–L3 DRGs or L4–L6 DRGs from each animal. Luciferase transcript levels of each condition were determined by RT-qPCR and normalized to the corresponding ones for the b-actin housekeeping transcript. Each value is then normalized to the mean of undersized L4–L6 values.

To evaluate whether the introduction of the stuffer fragment at the 3′ end of the transgene impacts its expression level, activity, and kinetics, we first monitored the luciferase reporter transgene expression in vitro following the infection of Vero cells with stuffed nrHSV-1 vectors at different multiplicity of infection (MOI) values compared to an undersized counterpart (Figure 4A). We detected comparable luciferase activity between both vector types at 24 h, 48 h, and 72 h post-infection at an identical MOI value (Figure 4A), indicating that both vectors can infect cells and induce robust transgene expression without differences in kinetics, independent of the presence of a stuffer DNA in proximity to the transgene.

For engineering purposes, the stuffer DNA sequence was inserted into the LAT region of the HSV-1 vector genome, positioned at the 3′ end of the transgene. This LAT region is known to remain open and transcribed in neuronal cells. To ensure that such insertion did not disrupt the properties of the HSV-1 LAT region, we explored the impact of introducing the stuffer DNA sequence into HSV-1 vectors on transgene expression in vivo in a mouse model. To that end, stuffed or undersized nrHSV-1 vectors expressing the luciferase reporter transgene were injected into the footpad of 7-week-old BALB/c mice. Following such injections, nrHSV-1 vectors can enter peripheral sensory neurons via their projections present in the footpad and travel along the afferent nerve fibers toward the spinal cord where it establishes latency predominantly in the lumbar dorsal root ganglia (DRG) L4 to L6, which innervate the lower leg [25]. DRG samples collected at 28 days post-injection were tested for luciferase transcript levels by RT-qPCR. Comparable transgene expression levels were found in L1–L3 and L4–L6 DRG between both vectors (Figure 4B). Additionally, higher levels of luciferase transgene were observed in L4–L6 DRG samples for both vectors compared to the L1–L3 DRG samples. These data confirm that the insertion of a stuffer DNA at the 3′ end of a LAT-inserted transgene had no adverse effects on effective delivery, capsid retrograde transport, and transgene expression in the target tissue.

## 3. Discussion

### 3.1. Genomic Instability of Small Non-Replicative HSV-1 Vectors

Our findings suggest that a reduction in the nrHSV-1 vector’s genome size correlates with increased genomic instability, which is consistent with previous studies and demonstrates a direct link between a smaller vector size and the higher occurrence of genomic rearrangements in other viral systems. For instance, in helper-dependent adenovirus vectors, genomes smaller than ~27 kb (75% of the AdWT genome) were shown to spontaneously rearrange to increase in genome size [2,6,7,26].

We observed that when the nrHSV1 genome size was reduced by more than 6% (9 kbp), viral populations exhibited significant genomic rearrangements over serial passages. These rearrangements led to the spontaneous formation of amplicons, which contained key regions such as the origin of viral DNA replication (oriS) and packaging (pac) signal, as well as genomic duplications originating from the first region. Moreover, the high frequency of amplicons points to a mechanism of genomic instability potentially driven by the suboptimal packaging size of the vector, which could limit its ability to stably package and propagate its genome.

Interestingly, we observed that stuffed nrHSV1 reached improved levels of productivity compared to the undersized nrHSV1. It has been previously shown that herpes virus genome over-lengthening led to spontaneous deletions and altered genome integrity [27]. Similarly, deletion of >15% of the guinea pig cytomegalovirus (GPCMV) wild-type genome length was also shown to lead to partial genome duplication as a compensatory mechanism for the suboptimal genome size [27]. In contrast, GPCMV mutants with larger deletions (13%) exhibited reduced replication efficiency [27]. This decrease was attributed to the loss of key viral genes rather than the reduction in genome size. However, smaller deletions of approximately 10% in both GPCMV and murine cytomegalovirus (MCMV) did not alter viral replication efficiency [27,28], suggesting that such viruses can accommodate variation in genome size up to 10%.

To our knowledge, no in-depth genome characterization has yet been performed on deletion-mutant herpes viruses in prior studies, likely due to the large size and repeated sequences of the viral genome prohibiting the effective use of Illumina short-read sequencing. In this study, we characterized the nrHSV1 genome using long-read sequencing for the first time. We demonstrated that as the nrHSV1 vector underwent successive passages, the genome size progressively increased, highlighting the importance of genome size in maintaining viral genome stability. This progressive enlargement, along with the associated instability, suggests a narrow window for effective vector design. Deviating too far from the wild-type genome’s size leads to genomic rearrangements and compromised vector homogeneity, which in turn hinders the reliable production of consistent viral stocks.

### 3.2. Restoration of nrHSV1 Genomic Stability by Inserting a Stuffer DNA

To address the genomic instability associated with smaller vector genomes, we introduced a stuffer DNA fragment to restore the vector size closer to that of the wild-type HSV-1 genome. The stuffer DNA, derived from non-coding regions of the HSV-1 genome, was designed to ensure no open reading frames or promoter sequences, thus preventing unintended gene expression. Importantly, this modification resulted in a net genome size of approximately 153.5 kbp, very similar to that of wild-type HSV-1 (152 kbp), and long-read ONT sequencing confirmed the successful insertion of the stuffer DNA without introducing genomic instability. These findings indicate that the stuffer DNA served to restore the viral genome size while preventing the formation of genomic rearrangements like amplicons or duplications, which were prevalent in undersized vectors. The 1.5 kbp difference between the stuffed nrHSV-1 vector used in this study and the wild-type HSV-1 genome size indicates that there is nevertheless a permissive window around the wild-type genome size that allows for maintaining genome stability. The addition of the stuffer DNA sequence also improved nrHSV-1 vector stability across multiple passages, as evidenced by the absence of rearrangements and consistent genome size over up to 10 passages in complementing Vero 7b cells. This genomic stability suggests that the restored genome size provides an optimal balance for viral propagation, ensuring stability without inducing the rearrangements seen in smaller vectors.

### 3.3. Stuffer DNA Does Not Compromise Transgene Expression

The nature of the DNA sequence around the transgene locus may have a significant impact on transgene expression [21,29]. Our results showed that the insertion of the stuffer DNA sequence did not adversely affect transgene expression both in vitro and in vivo. In Vero cells, luciferase activity was comparable between undersized and stuffed nrHSV-1 vectors with and without the stuffer fragment, with no significant difference in transgene expression levels or kinetics. These data demonstrate that the insertion of the stuffer DNA did not disrupt the efficiency of transduction or alter the expression kinetics of the transgene, ensuring that the vector remains suitable for gene delivery applications.

In vivo studies further corroborated these findings since the vectors containing the stuffer DNA exhibited similar transgene expression levels as those without the stuffer when injected into BALB/c mice. Both vectors displayed significant transgene expression within the neurons of the dorsal root ganglia (DRG), innervating the site of administration 28 days post-injection, indicating that both vectors were capable of reaching the target tissue and persisting over time in a latent state. Thus, the addition of the stuffer DNA does not hinder the vector retrograde transport ability or affect transgene expression in the target tissue.

It was previously described that the nature of the stuffer DNA (e.g., eukaryotic vs. prokaryotic) could impact transgene expression in hdAd vectors. Insertion of stuffer DNA essentially from bacteriophage lambda was previously used to promote stable genome structure in helper-dependent adenovirus vectors [30,31]. However, such stuffer DNA was subjected to epigenetic regulations that quickly down-regulated the expression of the proximal transgene in hdAd vectors [31]. While we did not characterize epigenetic marks on the nrHSV-1 episome, the transgene was expressed up to 28 days in mice DRGs, suggesting that the presence of DNA stuffer in the proximity of the transgene does not impact the transgene chromatin structure.

## 4. Materials and Methods

### 4.1. Cell Culture

African-green monkey Vero-derived complementing cell line 7b [20] was maintained in Dulbecco’s modified eagle medium (DMEM, Thermofisher, Waltham, MA, USA), supplemented with 10% fetal bovine serum (FBS, Eurobio, Surrey, UK) and 500 µg/mL geneticin (Thermofisher, Waltham, MA, USA).

Non-complementing Vero cells were grown in serum-free medium OptiPRO SFM (Thermofisher, Waltham, MA, USA) supplemented with 4 mM glutamine (GlutaMAX, Thermofisher, Waltham, MA, USA). Neuro2a cells were cultured in Eagle’s Minimum Essential Medium (EMEM, ATCC, Manassas, VA, USA) supplemented with 10% FBS (Eurobio, Surrey, UK).

iCell GlutaNeurons (Fujifilm, Madison, WI, USA), a highly enriched population of human cortical glutamatergic neurons derived from an induced pluripotent stem cell (iPSC), were thawed and cultured according to the manufacturer’s instructions.

All cells were cultured at 37 °C in a humidified incubator with 5% CO_2_.

### 4.2. Non-Replicative HSV-1 BAC Engineering

The genome of the wild-type (WT) HSV-1 F strain was originally cloned into a bacterial artificial chromosome (BAC) and used to construct non-replicative vectors [32].

HSV-1 BAC engineering was performed by using the scarless Red recombination in electrocompetent GS1783 bacteria as described before [33]. BACs were isolated using the PureLink HiPure Plasmid Midiprep Kit (Thermofisher, Waltham, MA, USA) according to the manufacturer’s instructions. DNA pellets were resuspended in 100 µL of nuclease-free water (Thermofisher, Waltham, MA, USA) and the concentration of purified DNA was measured using the Qubit 4 fluorometer (Thermofisher, Waltham, MA, USA). A total of 50 µL of each BAC construct was transfected on 0.6 × 10^6^ Vero 7b cells in 6-well plates using 7.5 µL of Lipofectamine 3000 (Thermofisher, Waltham, MA, USA) in Opti-MEM media (Thermofisher, Waltham, MA, USA) as recommended by the manufacturer. After overnight incubation at 37 °C, transfection medium was replaced with 2 mL of DMEM + 10% FBS medium. Transfected cells were checked regularly under a microscope, and samples were collected after 10 to 14 days.

### 4.3. Viral Amplification

The production of nrHSV1 (non-replicative herpes simplex virus type 1) was carried out using Vero 7b cells, a cell line suitable for the propagation of HSV1. For in vitro analysis, Vero 7b cells were infected at a multiplicity of infection (MOI) of 0.03 plaque forming unit (PFU). After a 1-h-and-30-min incubation at RT under consent agitation, the infected cells were seeded in T150 cm^2^ culture flasks (Corning, New York, NY, USA) at a density of 1.3 × 10^5^ cells per cm^2^ and incubated for 3–4 days to allow for viral replication. For large-scale production of nrHSV1 for the animal study, Vero 7b cells were seeded in a CS10 cell stack (6360 cm^2^, Corning, New York, NY, USA) at a density of 2 × 10^5^ cells per cm^2^.

After 3–4 days, cells and supernatants were lysed using 0.45 M NaCl (Sigma-Aldrich, St Louis, MO, USA) and 100 µg/mL dextran sulfate (Sigma-Aldrich, Saint-Louis, MI, USA) medium (4 h, 37·°C). Cell lysates were collected, sonicated (Eppendorf, Hambourg, Germany), clarified by centrifugation (2000× *g*, 5 min, 4 °C), and filtered through a 0.45 µm nitrate cellulose membrane (ClearLine, Brumath, France). The viral solution was concentrated by high-speed centrifugation for 1 h at 21,000× *g*, 4 °C, or 1 h 30, 13,000× *g*, 4 °C (large-scale production) (Thermofisher, Waltham, MA, USA). Viral pellets were resuspended in vector storage buffer (20 mM Histidine, 50 mM NaCl, 150 mM Sucrose, pH 6.7) and stored at −80 °C in small aliquots.

### 4.4. Plaque Forming Unit (PFU)

Plaque assay was performed on 0.8 × 10^6^ Vero 7b in 6-well plates (Corning, Corning, NY, USA). Cells were infected with 500 µL of ten-fold serial dilutions of viral stock for 1 h with regular rocking. Viral inoculum was replaced with 2 mL of DMEM containing 2% FBS and 1% carboxylmethyl cellulose (CMC, Thermofisher, Waltham, MA, USA). Four days post-infection (pi), cells were fixed with 4% formaldehyde (Dutscher, Brumath, France) and plaques were revealed using an anti-gD HRP-conjugated antibody (Santa-Cruz, Dallas, TX, USA). All viral stocks produced in the T150 cm2 flask had an infectious titer ranging from 5 × 10^6^ PFU/mL to 5 × 10^7^ PFU/mL. The infectious titer of undersized nrHSV-1 and stuffed nrHSV-1 used for the animal study had an infectious viral titer of 1.8 × 10^10^ PFU/mL and 8.79 × 10^9^ PFU/mL, respectively.

### 4.5. Sequencing

BAC and vectors produced were verified by next-generation sequencing (NGS) according to the manufacturer’s protocol of the Oxford Nanopore rapid barcoding kit, using the Minion sequencing device (Oxford Nanopore Technology, Oxford, UK). Basecalling was performed using MinKNOW 5.3.6 software (high-accuracy mode, filtering out reads shorter than 200 bp or of an overall score lower than 9). Sequencing data were analyzed with Geneious Prime^®^ software, version 2025.0.3 using the “map to reference” function and the Minimap 2.24 mapping algorithm and the “Find variations/SNPs” function (minimum variant frequency: 0.75; maximum variant *p*-value: 10^−6^) to identify variants.

### 4.6. Viral Gene Copy

Viral DNA was extracted with the ISOLATE II Genomic DNA Kit (Bioline, London, UK) according to the manufacturer’s instructions. Quantitative (q) PCR was performed using the QS6 PRO PCR System (Thermofisher, Waltham, MA, USA) with customized primers and probe (Thermofisher, Waltham, MA, USA) specific for the ICP0, UL19, or the luciferase transgene gene. Viral genome copies were quantified by interpolation on a standard curve generated by a ten-fold serial dilution of a plasmid of known concentration.

### 4.7. Productivity Assay

Replicate wells of complementing Vero-7b seeded at a density of 2 × 10^5^ cells per well of a 24-well plate were infected at an MOI of 0.03 for 1 h at 37 °C with regular agitation and incubated at 37 °C and 5% CO_2_. Media were harvested daily and the infectious titer was determined by standard plaque assay on Vero 7b cells.

### 4.8. Toxicity

Non-complementing Vero and Neuro2a cells were seeded in a white 96-well plate (Nunc, Roskilde, Denmark) at a density of 4 × 10^4^ cells/well. Cells were infected at the indicated MOI values and cell viabilities were determined at indicated time points post-infection using the Celltiter Glo luminescent assay (Promega, Madison, WI, USA) as described by the manufacturer.

### 4.9. In Vitro Luciferase Transgene Activity

Vero cells were seeded in a white 96-well plate (Nunc) at a density of 4 ×10^4^ cells/well. Cells were infected at the indicated MOI values and luciferase activity was quantified at the indicated time point using the Bright-Glo Luciferase assay system (Promega, Madison, WI, USA). Luminescence was measured on the GloMax Discover plate reader (Promega, Madison, WI, USA).

### 4.10. In Vivo nrHSV1 Injection and Tissue Collection

Female BALB/c mice that were 7 weeks old were injected subcutaneously with buprenorphine (0.1 mg/kg) and Metacam (2 mg/kg) at least 20 min before nrHSV1 injection. Mice were anesthetized with a mix of isoflurane and oxygen and 1.76 × 10^8^ PFU of undersized-nrHSV1 or stuffed-nrHSV1 vectors were injected into the right footpad of each mouse using a 31 G needle pre-mounted on a disposable syringe. Non-infected animals received 20 µL of vehicle solution. Animals received a second subcutaneous injection of Metacam (2 mg/kg) 5 h, day 1 and day 2 post-injection. At day 28 post-injection, animals were euthanized under anesthesia by cervical dislocation. Ipsilateral (L1 to L6) DRGs were sampled, placed into cryovial tubes, and immediately frozen by immersion in liquid nitrogen.

### 4.11. Transgene Expression Quantification by qRT-PCR

For qRT-PCR, total DNA and RNA were extracted from pooled L1–L3 or L4–L6 DRGs using the Quick DNA/RNA Microprep plus kit (ZymoResearch, Irvine, CA, USA) and quantified using the Qubit 4 system (Thermofisher, Waltham, MA, USA). cDNA was synthesized using 250 ng of RNA and the SuperScript IV Reverse-transcriptase kit (Thermofisher, Waltham, MA, USA). Real-time PCR was performed in duplicate using the QS6 PRO Real-Time PCR System (Thermofisher, Waltham, MA, USA). Results of b-actin housekeeping mRNA were used to normalize the data. Each value was then normalized to the undersized L4-L6 average value. All qRT-PCR primers used in this study are listed in Table S1.

## 5. Conclusions

This study highlights for the first time the critical role of genome size in maintaining the stability of nrHSV-1 vectors. We demonstrate that reducing the genome size of nrHSV-1 vectors beyond a certain threshold, approximately 5% of the wild-type size, results in genomic instability, compromising vector productivity, homogeneity, and stability. By introducing a stuffer DNA fragment with appropriate characteristics, we successfully restored the vector genome to the same size as the wild type, which significantly improved genomic stability and viral productivity without impacting transgene expression. The restoration of the genome’s size creates optimal conditions for efficient packaging and stable propagation of the vector, thereby enhancing its potential for gene therapy applications. Hence, our results underscore the importance of maintaining the vector genome’s size for achieving desirable performance characteristics in gene delivery systems, paving the way for innovative advancements in the field.

## Data Availability

All relevant data supporting the findings of this study are included in the manuscript and its Supplementary Materials. Additional datasets are available from the corresponding author upon reasonable request.

## Supplementary Materials

The following supporting information can be downloaded at https://www.mdpi.com/article/10.3390/ijms26104941/s1.
