# Supplementary material for: Genomic Size Is Critical to Guarantee the Genomic Stability of Non-Replicative HSV1 Vectors
Source: Int J Mol Sci. 2025 May 21;26(10):4941. doi: 10.3390/ijms26104941 (PMC12111873; doi:10.3390/ijms26104941)
Supplement: Supplementary file 1 [file ijms-26-04941-s001.zip › ijms-3574884-supplementary-figures.pdf]

Supplementary Figures

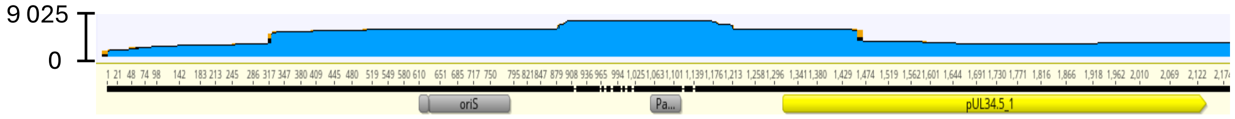

Supplementary Figure S1: Representative ONT sequencing of amplicons sequences. The coverage is represented as a blue line with read depth (measured in the number of reads per base) plotted along the y-axis. A higher coverage is observed in the genomic region containing oriS and pac signals indicating that such sequences are present in a higher proportion.

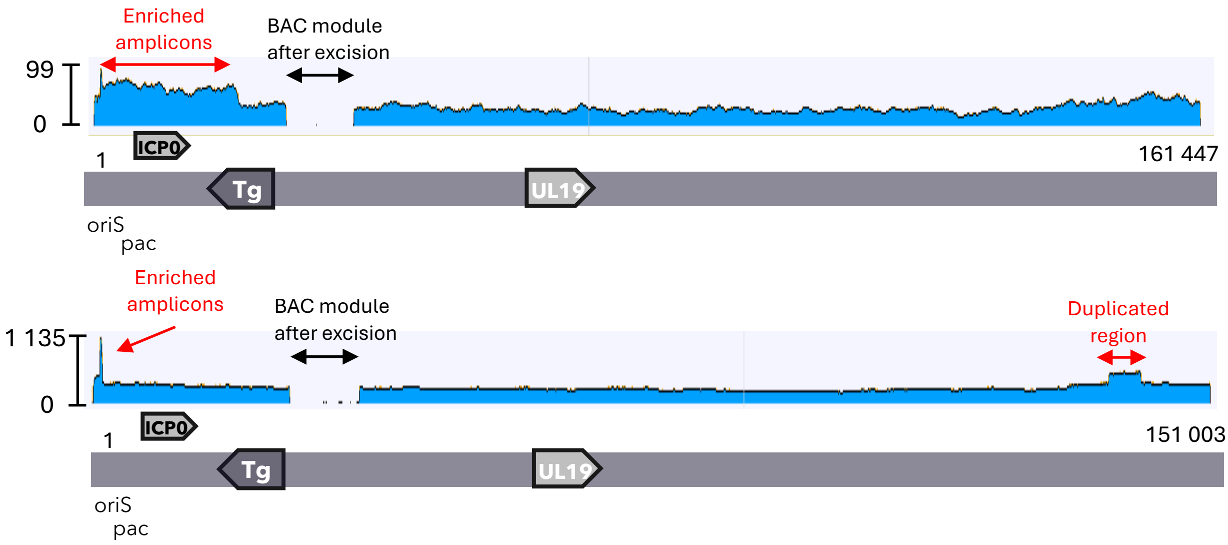

Supplementary Figure S 2: Representative Oxford Nanopore Technology sequencing of undersized nrHSV1 vector. Genomic DNA were extracted from purified vector stocks. The coverage is represented as a blue line with read depth (measured in the number of reads per base) plotted along the y-axis. Amplicons enriched regions and duplicated regions are shown by red arrows. Localization of relevant genomic elements are shown (origin of replication (oriS), packaging signal (pac), ICP0, transgene (Tg), UL19 gene)

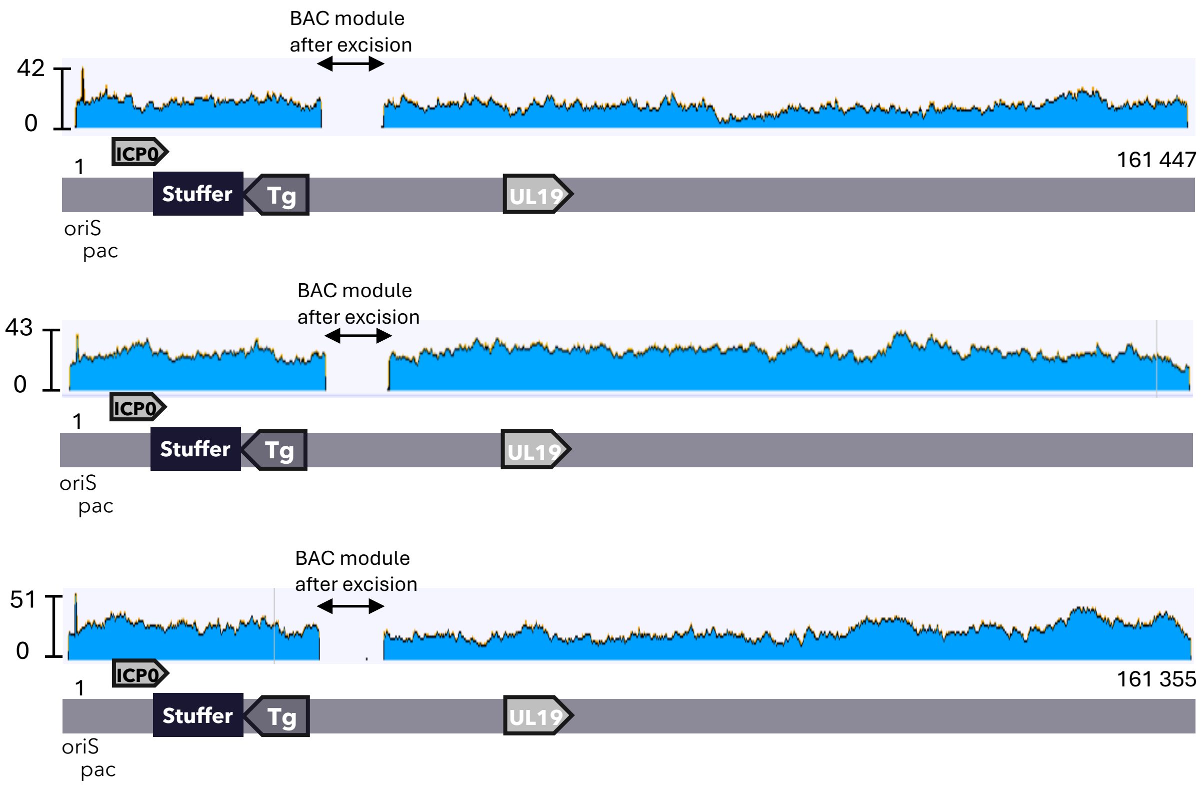

Supplementary Figure S3: Representative Oxford Nanopore Technology sequencing of the stuffed nrHSV1 vector. Genomic DNA were extracted from purified vector stocks. The coverage is represented as a blue line with read depth (measured in the number of reads per base) plotted along the y-axis. Localization of relevant genomic elements are shown (origin of replication (OriS), packaging signal (pac), ICP0, stuffer DNA, transgene (Tg), UL19 gene)
